# Distinct Roles for the N- and C-terminal Regions of M-Sec in Plasma Membrane Deformation during Tunneling Nanotube Formation

**DOI:** 10.1038/srep33548

**Published:** 2016-09-15

**Authors:** Shunsuke Kimura, Masami Yamashita, Megumi Yamakami-Kimura, Yusuke Sato, Atsushi Yamagata, Yoshihiro Kobashigawa, Fuyuhiko Inagaki, Takako Amada, Koji Hase, Toshihiko Iwanaga, Hiroshi Ohno, Shuya Fukai

**Affiliations:** 1 Laboratory of Histology and Cytology, Graduate School of Medicine, Hokkaido University, North 15, West 7, Kita-ku, Sapporo 060-8638, Japan; 2 Laboratory for Intestinal Ecosystem, RIKEN Center for Integrative Medical Sciences (IMS), 1-7-22 Suehirocho, Tsurumi, Yokohama, Kanagawa 230-0045, Japan; 3 Structural Biology Laboratory, Life Science Division, Synchrotron Radiation Research Organization and Institute of Molecular and Cellular Biosciences, The University of Tokyo, Tokyo 113-0032, Japan; 4 Department of Medical Genome Sciences, Graduate School of Frontier Sciences, The University of Tokyo, Chiba 277-8501, Japan; 5 Department of Structural Biology, Faculty of Advanced Life Science, Hokkaido University, N-21, W-11, Kita-ku, Sapporo 001-0021, Japan; 6 Division of Biochemistry, Keio University Faculty of Pharmacy, 1-5-30 Shibakoen, Minato-ku, Tokyo 105-8512, Japan; 7 Division of Immunobiology, Department of Medical Life Science, Graduate School of Medical Life Science, Yokohama City University, 1-7-29 Suehiro, Tsurumi, Yokohama 230-0045, Japan; 8 CREST, JST, Saitama 332-0012, Japan

## Abstract

The tunneling nanotube (TNT) is a structure used for intercellular communication, and is a thin membrane protrusion mediating transport of various signaling molecules and cellular components. M-Sec has potent membrane deformation ability and induces TNT formation in cooperation with the Ral/exocyst complex. Here, we show that the N-terminal polybasic region of M-Sec directly binds phosphatidylinositol (4,5)-bisphosphate for its localization to the plasma membrane during the initial stage of TNT formation. We further report a crystal structure of M-Sec, which consists of helix bundles arranged in a straight rod-like shape, similar to the membrane tethering complex subunits. A positively charged surface in the C-terminal domains is required for M-Sec interaction with active RalA to extend the plasma membrane protrusions. Our results suggest that the membrane-associated M-Sec recruits active RalA, which directs the exocyst complex to form TNTs.

Extension of the cell surface occurs during the early stages of cell morphogenesis, migration and engulfment and generates protruded membrane structures such as lamellipodia, pseudopodia, filopodia and microvilli^1^. Tunneling nanotubes (TNTs; also referred to as membrane nanotubes) are a relatively recently recognized membrane structure and are thin protrusions of the plasma membrane that physically connect remote cells, providing continuity between them^2^. Cells of various types can form TNTs for intercellular communication between the connected cells. Among immune cells, antigen exposure induces intercellular calcium fluxes via TNTs in myeloid lineage cells including dendritic cells and macrophages^3^. In natural killer (NK) cells, lytic granules can be delivered via TNTs to lytic synapses that are formed between the distal ends of the TNTs and distant target cells^4^. TNTs in most cell types contain actin filaments and lack microtubules, however NK cells contain microtubules, likely for the delivery of lytic granules, which depend on microtubules for trafficking. Remarkably, TNTs can transfer large membranous components such as transport vesicles and organelles in a cell-type-specific manner^5^,^6^. Moreover, TNT and TNT-related structures are thought to facilitate intercellular spreading of viruses and pathogenic proteins^7^,^8^. Thus, morphological and functional features of TNTs have been extensively investigated. However, the identity of molecules involved in the membrane protrusion of TNTs had not been substantially elucidated.

We recently discovered that M-Sec, a 73-kDa cytosolic protein also known as tumor necrosis factor alpha-induced protein 2 (TNFaip2) or B94^9^, plays a central role in inducing membrane protrusions during TNT formation^10^. Depletion of M-Sec by RNA interference (RNAi) significantly reduces TNT formation and TNT-mediated intercellular propagation of calcium flux in a macrophage cell line, Raw264.7. Conversely, forced expression of M-Sec in HeLa cells induces numerous membrane protrusions, some of which fuse with the plasma membrane of remote cells to form TNTs and allow calcium flux to propagate between the connected cells. Constitutive expression of M-Sec is essentially restricted to cells of myelomonocytic lineage (*e.g.* dendritic cells and macrophages)^11^ and some epithelial cells in normal tissues (*e.g.* M cells in the intestine)^10^, whereas M-Sec expression in these cells as well as in other lineages can be upregulated under conditions known to enhance TNT formation. For example, TNFα^9^ and lipopolysaccharide (LPS)^10^, which have been reported to be TNT-inducing factors^12^, enhance the expression of M-Sec. Treatment of rat hippocampal astrocytes with hydrogen peroxide increases the expression of M-Sec mRNA, resulting in TNT formation^13^. Infection with human T-cell leukemia virus type 1 (HTLV-1), which increases TNT-like cellular conduits, also induces M-Sec mRNA expression in T cells^14^.

M-Sec-induced TNT formation is accompanied by actin cytoskeleton remodeling, which requires the association with RalA, a small GTPase, and its downstream effector, the exocyst complex^10^. RNAi-mediated depletion of RalA or the exocyst subunits, Sec5 or Sec6, significantly impairs M-Sec-induced membrane extension. Overexpression of mutant RalA48W or RalA38R that cannot bind to the exocyst subunits, Exo84 or Sec5, respectively, significantly reduces M-Sec-induced TNT formation^10^,^15^. The leukocyte-specific transcript 1 protein (LST1) is also reportedly associated with TNT formation^15^. Ectopic expression of Lst1 induces TNT-like membrane protrusions. Lst1 is a single-pass membrane protein and interacts with the RalA/exocyst complex. Therefore, Lst1 likely serves as a scaffold on the plasma membrane to facilitate the association of molecules that control TNT formation.

M-Sec interacts with an active form of RalA and Lst1^10^,^15^, suggesting that it induces TNT formation with the aid of the RalA/exocyst complex and Lst1. Moreover, M-Sec has a strong membrane deformation capability for inducing TNT-like plasma membrane protrusions. M-Sec is therefore recognized as the key for understanding molecular mechanisms of TNT formation, which, however, have remained elusive. In this study, we first defined the important functional sites in M-Sec by deletion and mutation analysis. We found that M-Sec binds phosphoinositides on the plasma membrane via its N-terminal polybasic region. Second, we determined the crystal structure of the near-full-length M-Sec at 3.0 Å resolution. The M-Sec structure has a significant similarity to subunits of membrane tethering complexes, including the exocyst complex^16^,^17^,^18^,^19^, the Dsl1 complex^20^, the conserved oligomeric Golgi (COG) complex^21^ and the Golgi-associated retrograde protein (GARP) complex^22^,^23^. We identified a conserved, positively charged surface in the C-terminal half that is essential for M-Sec interaction with active RalA and promotion of TNT formation by extending out the plasma membrane. Our study reveals molecular mechanisms underlying TNT formation and further provides new insight into the relationship between M-Sec structure and its function.

## Results

### N- and C-terminal regions of M-Sec play distinct roles in TNT formation

M-Sec has sequence similarity to Sec6 (human M-Sec shows 23% amino acid identity and 46% similarity to rat Sec6 [GenBank accession number NP_001020135])^24^. However, the functional domains or regions of M-Sec are totally unknown. Therefore, we first attempted to identify the regions in M-Sec that are important for TNT formation. We have previously shown that transient expression of M-Sec in HeLa cells can induce membrane protrusions extending out from the plasma membrane, some of which tether onto remote cells to form TNT-like structures^10^. We thus reasoned that transient expression of M-Sec mutants would be a useful method to examine the regions in M-Sec required for inducing the membrane protrusions during TNT formation.

**Figure 1:**
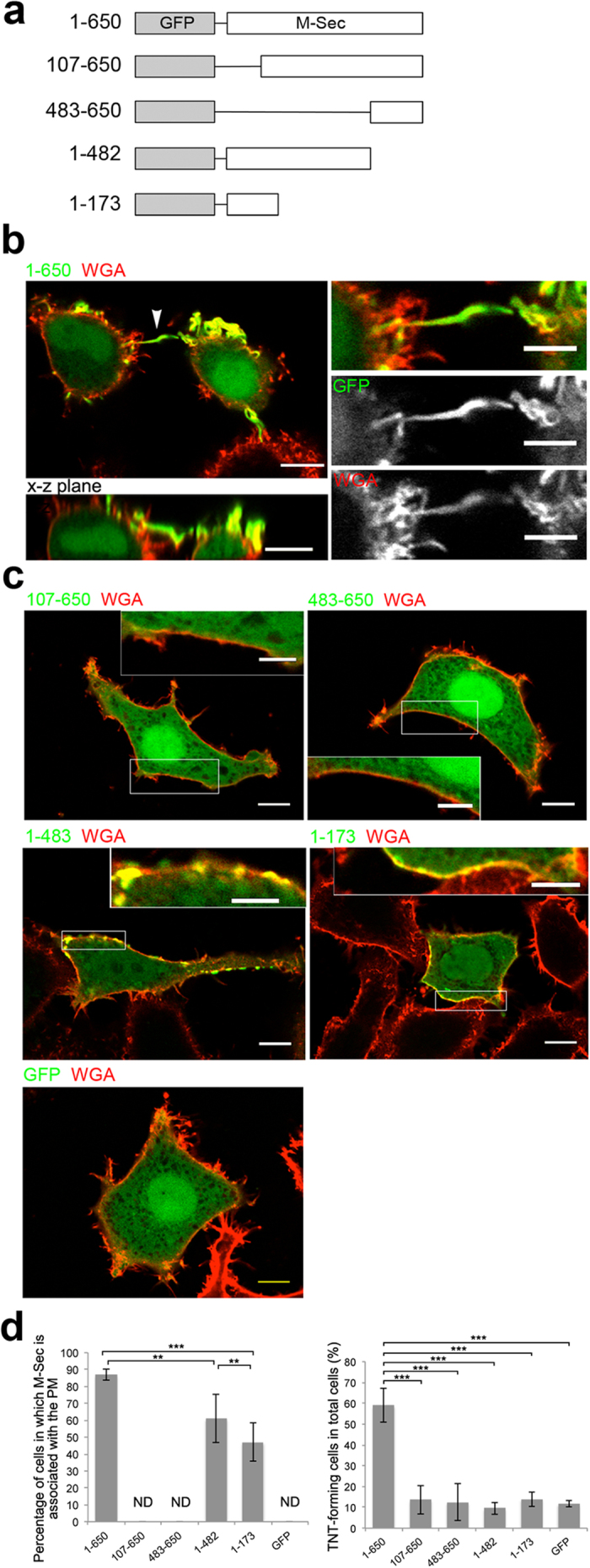
The N-terminal region of M-Sec is responsible for its recruitment to the plasma membrane and the C-terminal region is required for formation of protruding membrane extensions. (**a**) Diagrams of the full-length and truncated M-Sec proteins. Each protein name indicates the amino-acid residue number: 1–650 is the full-length M-Sec protein, 107–650 is the truncated protein consisting of amino-acid residues 107 to 650, and so on. GFP: Green fluorescent protein. (**b**,**c**) HeLa cells were transfected with expression vectors encoding the full-length (**b**) or truncated (**c**) M-Sec proteins fused with GFP. Twenty-four hours after transfection, the cells were treated with WGA-lectin at 4 °C for labeling the plasma membrane and then fixed. (**b**) Arrowhead indicates a M-Sec-induced membrane protrusion shown in the x-z plane in the lower panel. Right panels are highly magnified images of the membrane protrusion. Scale bars represent 10 μm in the left panels and 5 μm in the right panels. (**c**) The inserted image in each panel is the highly magnified image of boxed area. (**d**) Quantification of cells in which M-Sec colocalized with WGA (the left panel) and TNT-forming cells in total cells (the right panel). TNT-forming cells are cells connected with two or more cells by WGA-positive thin membrane structures that do not touch the substratum. Data are presented as the mean ± s.d. and approximately 100 cells per group were analyzed. **P < 0.05, ***P < 0.001; *P* value is calculated by Tukey-Kramer’s tests. ND: not detected.

We constructed N- and C-terminal deletion mutants of GFP-tagged M-Sec as shown in Fig. 1a, and expressed them in HeLa cells. Consistent with our previous observation^10^, the full-length GFP-M-Sec (residues 1–650) induced the formation of a number of membrane protrusions, whose location coincided with that of GFP-M-Sec (Fig. 1b). The position and size of the tags added to M-Sec have essentially no effect on its function; transfection of the C-terminally GFP-tagged M-Sec gave similar results to that of the C-terminally HA- or N-terminally GFP-tagged M-Sec (ref. 10). When the N-terminally deleted M-Sec constructs (residues 107–650 and 483–650) were expressed in HeLa cells, the mutant proteins diffused throughout the cytoplasm without inducing the GFP-positive membrane protrusions. On the other hand, the C-terminally deleted M-Sec mutants (residues 1–482 and 1–173) were correctly localized to the plasma membrane, but still failed to induce the GFP-positive membrane protrusions (Fig. 1c,d). These results indicate that induction of membrane protrusions for TNT formation requires both the N-terminal and C-terminal regions of M-Sec. M-Sec localization to the plasma membrane, mediated by its N-terminal region, is prerequisite for its function, while the C-terminal region probably functions in a subsequent step during TNT formation.

### The N-terminal polybasic region of M-Sec is responsible for its interaction with lipids and formation of membrane nanotubes

**Figure 2:**
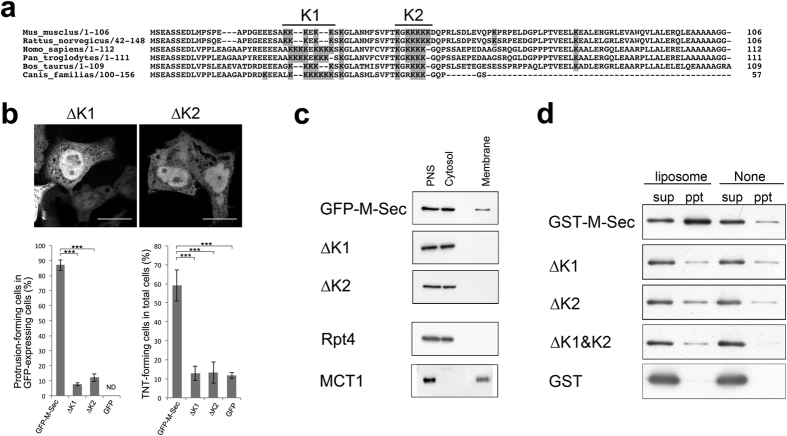
The lysine-rich regions in the N-terminal domain of M-Sec are required for direct interaction with membrane lipids. (**a**) Amino acid sequence alignment of the N-terminal region of M-Sec. Amino acid sequences from six mammalian species were aligned by ClustalX software (version 2.0.10; www.clustal.org/clustal2)^60^. Lysine residues are highlighted by gray backgrounds, and two conserved lysine-rich regions (residues 24–29 and 42–48) are termed K1 and K2, respectively. (**b**) HeLa cells were transfected with expression vectors encoding GFP-tagged ∆K1 or ∆K2 protein, which lacks the K1 or K2 region shown in (**a**), respectively. Fluorescent microscopic analysis was performed and TNT-forming cells were quantified as in Fig. 1. Protrusion-forming cells are cells that have GFP-positive membrane protrusions (including the TNT structure as well as a membrane protrusion of which the edge is free and does not connect with another cell). Data are presented as the mean ± s.d. and approximately 100 cells per group were analyzed. ***P < 0.001; *P* value is calculated by Tukey-Kramer’s tests. ND: not detected. Scale bars represent 10 μm. (**c**) HeLa cells were transfected with expression vectors encoding GFP-M-Sec, GFP-∆K1 or GFP-∆K2. Cell fractionation and western blotting analysis were performed as described in *Methods*. PNS stands for postnuclear supernatant. Monocarboxylate transporter 1 (MCT1) and Rpt4 were used as membrane and cytosolic fraction markers, respectively. (**d**) The indicated GST-tagged proteins were incubated with or without liposomes made with total liver lipids. The proteins co-precipitated with liposomes (ppt) and in the supernatant (sup) were visualized by Western blotting using anti-GST antibody. ∆K1&K2 indicates M-Sec protein lacking both K1 and K2.

Alignment of N-terminal amino acid sequences of M-Sec from six mammalian species revealed a highly conserved polybasic region comprised of two lysine clusters (Fig. 2a). In mouse M-Sec, the first and second clusters (named K1 and K2, respectively) comprise residues 24–29 and 42–49, respectively. Polybasic regions are known to facilitate the recruitment of many proteins to the plasma membrane^25^. To assess the function of the N-terminal polybasic region of M-Sec, we generated GFP-tagged M-Sec mutants that lack K1 and K2 (∆K1 and ∆K2, respectively). When these mutants were transiently expressed in HeLa cells, both ∆K1 and ∆K2 proteins diffused throughout the cytoplasm and neither could induce the GFP-positive membrane protrusions (Fig. 2b). Cell fractionation analyses (see *Methods*) showed similar results for their localization. Wild-type GFP-M-Sec expressed in HeLa cells was detected in both membrane and cytosolic fractions, whereas ∆K1 and ∆K2 mutants were detected only in the cytosolic fraction (Fig. 2c). We next tested lipid binding activity of wild-type M-Sec and ∆K mutants by *in vitro* co-sedimentation assays using unilamellar liposomes prepared from liver total lipids (Fig. 2d). After ultracentrifugation, the wild-type M-Sec was precipitated due to its interaction with the liposomes, but ∆K1, ∆K2 or their combination mutant (∆K1&K2) were not.

### M-Sec binds phosphoinositides via the polybasic region

**Figure 3:**
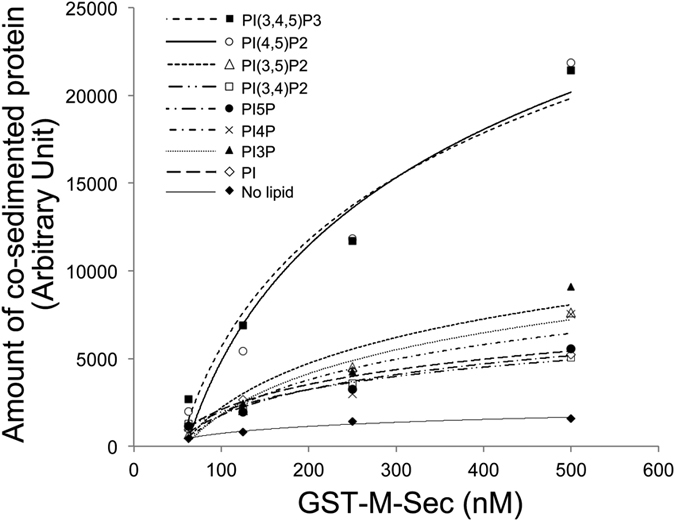
Binding of M-Sec with PI(4,5)P_2_ and PI(3,4,5)P_3_. GST-M-Sec protein was incubated with liposomes containing 20% phosphatidylethanolamine, 30% phosphatidylserine and 45% phosphatidylcholine supplemented with 5% phosphatidylinositol as indicated in the graph. The proteins co-precipitated with liposomes were visualized by Coomassie Brilliant Blue staining. The bound proteins were quantified by densitometry. The data is a representative of three independent experiments.

To examine which types of lipids bind to the polybasic region of M-Sec, we prepared unilamellar liposomes composed of phosphatidylcholine, phosphatidylethanolamine and phosphatidylserine supplemented with respective phosphatidylinositols, and performed lipid co-sedimentation assays. M-Sec preferentially co-precipitated with liposomes containing phosphatidylinositol (4,5)-bisphosphate [PI(4,5)P_2_] or phosphatidylinositol (3,4,5)-trisphosphate [PI(3,4,5)P_3_] (Fig. 3). In addition, ∆K1&K2 did not co-sediment with liposomes containing PI(4,5)P_2_ (Supplementary Fig. S1). These results suggest that M-Sec is localized to the plasma membrane via direct interactions of the polybasic region with PI(4,5)P_2_ and/or PI(3,4,5)P_3_ on the plasma membrane.

### PIP2 is localized on M-Sec-induced membrane nanotubes

**Figure 4:**
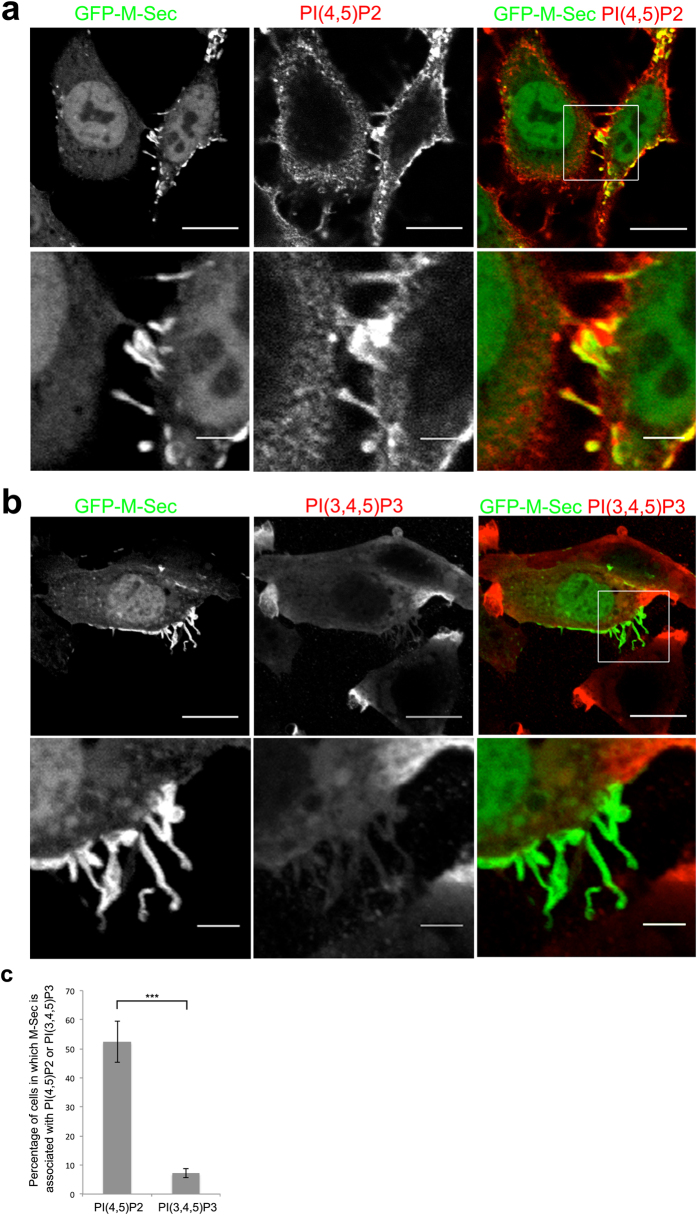
PI(4,5)P_2_ is localized in the M-Sec-induced membrane extensions. (**a**,**b**) HeLa cells were transfected with an expression vector encoding GFP-M-Sec. Twenty-four hours after transfection, the cells were fixed and then subjected to immunocytochemistry by using anti-PI(4,5)P_2_ (**a**) or PI(3,4,5)P_3_ (**b**) antibodies. The right color panels show merged images. The lower panels show the enlarged images of the boxed region in the upper panels. Scale bars represent 20 and 5 μm in the upper and lower panels, respectively. (**c**) The proportion of cells in which GFP-M-Sec is associated with PI(4,5)P_2_ or PI(3,4,5)P_3_ to total GFP-expressing cells. Data are presented as the mean ± s.d. from three independent experiments and approximately 200 cells per group were analyzed in each experiment. ***P < 0.001; *P* value is calculated by Student’s *t*-test.

Our observations so far suggest that PI(4,5)P_2_ and/or PI(3,4,5)P_3_ are associated with M-Sec-induced membrane nanotube formation. Thus, we examined distributions of PIP2 and PIP3 in HeLa cells expressing GFP-M-Sec by immunocytochemistry using monoclonal antibodies against PI(4,5)P_2_ and PI(3,4,5)P_3_. We found that endogenous PI(4,5)P_2_ was localized on the plasma membrane and accumulated at sites of GFP-M-Sec positive membrane protrusion formation (Fig. 4a). In contrast, PI(3,4,5)P_3_ was enriched in the plasma membrane ruffles at the cell periphery as described previously^26^, but not in the GFP-M-Sec positive membrane protrusions (Fig. 4b). Similar results were obtained using PI(4,5)P_2_- and PI(3,4,5)P_3_-specific probes: GFP-tagged pleckstrin homology (PH) domain of the phospholipase Cδ (PLCδ-PH-GFP) for PI(4,5)P_2_^27^ and the PH domain of the serine/threonine-protein kinase Akt (Akt-PH-GFP) for PI(3,4,5)P_3_^28^ (Supplementary Fig. S2). PLCδ-PH-GFP but not Akt-PH-GFP was localized to M-Sec-induced membrane protrusions. We further confirmed that PI(4,5)P_2_ is localized on TNT structures in Raw264.7 cells (Supplementary Fig. S3).

### Crystal Structure of M-Sec

**Figure 5:**
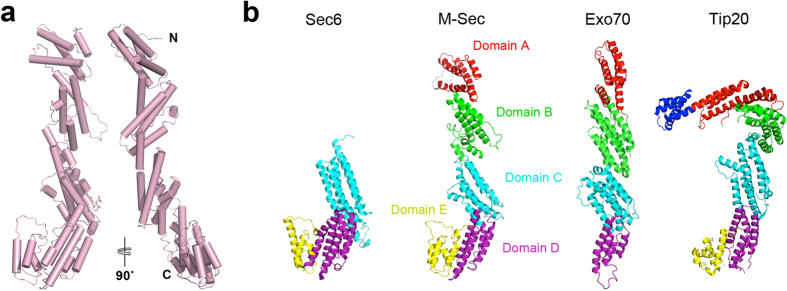
Crystal structure of mouse M-Sec. (**a**) Crystal structure of nearly full-length M-Sec (residues 67–650). The front and side views are shown as cartoon models. (**b**) Structural comparison of mouse M-Sec with yeast Sec6 (PDB 2FJI, residues 411–805 out of 805), Exo70 (PDB 2B7M, residues 67–623 out of 623) and Tip20 (PDB 3FHN, residues 1–701 out of 701), based on pairwise alignments. Sec6, Exo70 or Tip20 structures were matched to the M-Sec structure using the program DaliLite with Z score of 25.1, 11.4 and 12.8, respectively. Domains A, B, C, D and E are colored red, green, cyan, purple and yellow, respectively. The N-terminal region of Tip20 is colored blue.

To obtain high resolution M-Sec structural information that might provide insight into the mechanism of membrane protrusion formation, we determined the crystal structure of the N-terminally truncated (residues 1–66) M-Sec at 3.0 Å resolution (Supplementary Table S2). Although we could also crystallize the full-length M-Sec, the N-terminal 66 residues are disordered and the corresponding electron density could not be observed. The structure consists entirely of α-helices and intervening loops of variable length, organized into a series of helix-bundle domains (Fig. 5a). This domain structure is similar to that of subunits of multi-subunit membrane tethering complexes (Exo70^17^,^19^, Exo84^16^, Sec6^18^, and Sec15^29^ of the exocyst complex, Tip20 and Dsl1 of the Dsl1 complex^20^,^30^, Cog4 of the COG complex^21^, and Vps53 and Vps54 of the GARP complex^22^,^23^) and cargo-binding domains of myosin V-family proteins^31^. Particularly, there is a remarkable resemblance between the C-terminal halves of M-Sec and yeast Sec6^18^ (Fig. 5b) (Cα r.m.s.d of 3.0 Å over 278 amino acids), as expected from their high sequence similarity.

Crystal structures of yeast Tip20 and mouse and yeast Exo70 subunits have been reported as full-length or near-full-length proteins (lacking the N-terminal 60 or 84 residues out of total 623 or 653 residues in yeast and mouse Exo70 structures, respectively)^16^,^17^,^19^,^20^. Exo70 and Tip20 share a similar core architecture consisting of helix-bundle domains^16^,^20^ (domains A-D; Fig. 5b). However, their overall shapes are different from each other. Domains A to D of Exo70 are arranged in a linear array, forming an overall straight rod-like shape, whereas those of Tip20 are kinked at the junction between domains B and C, forming an overall hooked shape (Fig. 5b and Supplementary Fig. S4). The domain arrangement of M-Sec (domain A: Helix 1–4, domain B: Helix5–9, domain C: Helix9–13, domain D: Helix13–16) is similar to that of Exo70 and adopts a straight rod-like conformation. In addition, M-Sec has an extra C-terminal domain (domain E: Helix 17–20), which is conserved among tethering subunits and cargo-binding domains of myosin V (Supplementary Fig. S4)^18^,^20^,^21^,^23^,^30^,^31^,^32^.

### The positively charged surface in the C-terminal half of M-Sec is responsible for binding to active RalA and extension of membrane nanotubes

**Figure 6:**
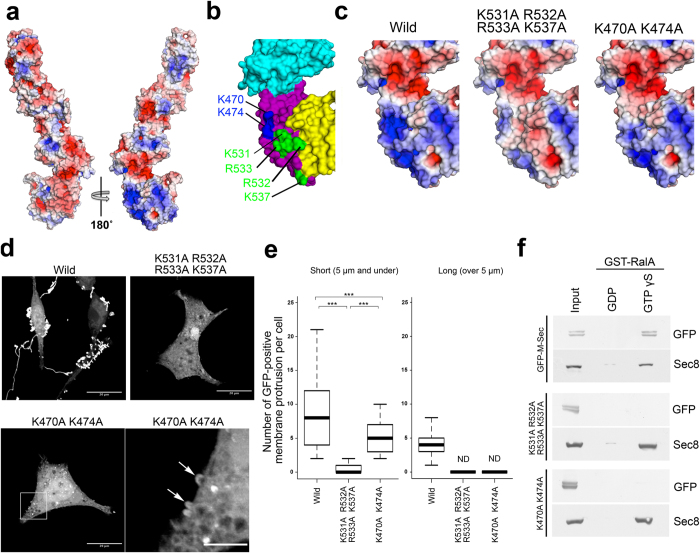
The positively charged surface in the C-terminal region of M-Sec is required for inducing long membrane protrusions. (**a**) Electrostatic surface potential of M-Sec on a scale from −5 *kT*/*e* (red) to +5 *kT*/*e* (blue). The electrostatic calculation was performed using the program APBS tool and visualized by PyMOL. (**b**) Representation of the six basic amino-acid residues clustered on a positively charged surface on the C-terminal region. K531, R532, R533, and K537 are colored blue, while K470 and K474 are colored green. Domains C, D and E are colored cyan, purple and yellow, respectively. (**c**) Electrostatic surface potentials calculated from model structures of the quadruple mutant K531A/R532A/R533A/K537A and the double mutant K470A/K474A. (**d**) Fluorescent microscopic images of HeLa cells expressing GFP-M-Sec or the quadruple or double mutant. The lower right panel is the enlarged image from the boxed area in the lower left panel. Arrows indicate short membrane protrusions. The scale bar in the lower right panel represents 5 μm, while those in other panels represent 20 μm. (**e**) Boxplots of the number of GFP-positive membrane protrusions per cells. The length of each protrusion was measured by ImageJ software. Approximately 100 cells per group were analyzed. ***P < 0.001; *P* value is calculated by Tukey-Kramer’s tests. ND: not detected (**f**) GST-pull down assays using the purified GST-RalA protein and HEK293A cell lysates containing the indicated proteins. Each input is 2% equivalent volume of the lysate before GST-pull down.

M-Sec 1–482, which mostly lacks domains D and E, cannot induce membrane protrusions (Fig. 1b). Based on this observation, together with the structural features of these domains revealed by our M-Sec crystal structure, we hypothesized that these two domains are functionally important for membrane protrusion formation. The calculated electrostatic surface potential of M-Sec (Fig. 6a,c) revealed a positively charged surface that covers both domains D and E, where conserved lysine and arginine residues are widely distributed and exposed to solvent (Fig. 6b and Supplementary Fig. S5a). We speculated that this positively charged surface might be associated with the formation of the membrane protrusions. To test this possibility, we introduced two sets of mutations that would decrease the overall positive charge in domains D and E: One set is a quadruple mutation K531A/R532A/R533A/K537A, and the other is a double mutation K470A/K474A (Fig. 6b,c). Both mutants retained the plasma membrane localization, as shown by cell fractionation assays (Supplementary Fig. S5b), but failed to induce the long GFP-positive membrane protrusions (over 5 μm; Fig. 6d,e). *In vitro* lipid co-sedimentation assays further showed that binding of M-Sec to PI(4,5)P_2_ was not affected by these mutations (Supplementary Fig. S1), suggesting that domain D or E is not responsible for binding to membrane lipids.

We previously showed that purified active GST-RalA could pull-down ectopically expressed HA-M-Sec from cell extracts^10^. Knockdown of RalA or overexpression of its dominant-negative form, RalA28N, abrogated the induction of long membrane nanotubes by M-Sec, although a limited number of immature, small membrane deformations were observed. These observations are entirely consistent with the results from K531A/R532A/R533A/K537A and K470A/K474A M-Sec mutations (Fig. 6d). Thus, we speculated that the positively charged surface on domains D and E is responsible for the interaction with RalA. To test this possibility, we examined the binding of purified GST-RalA to the quadruple or double M-Sec mutants expressed in HEK293 cells by GST pull-down assay (Fig. 6e). Consistent with previous reports, wild-type M-Sec bound to RalA in a GTP-dependent manner^10^,^15^. On the other hand, no binding of either M-Sec mutant to RalA was detected in the presence or absence of GTPγS or GDP, while the interaction of Sec8 with RalA in the presence of GTPγS was unaffected. These results suggest that the positively charged surface of domains D and E is responsible for the interaction with RalA, which is required for subsequent extension of the plasma membrane.

## Discussion

In this study, we combined mutational and crystallographic analyses to define the functional and structural characteristics of the N- and C-terminal regions of M-Sec in TNT formation. Based on our present observations, we propose the following stepwise process of M-Sec-induced TNT-formation: Cytosolic M-Sec is recruited to the plasma membrane through direct binding to PI(4,5)P_2_ and/or PI(3,4,5)P_3_. Subsequently, the membrane-bound M-Sec recruits active RalA via the positively charged surface patch on domain D and E at its C-terminus, which elicits membrane deformation and promotes the subsequent formation of membrane extensions in cooperation with the exocyst complex and Lst1. The exocyst complex and Lst1 reside on the plasma membrane by PI(4,5)P_2_-binding of Exo70 and Sec3 subunits^33^,^34^,^35^,^36^ and the transmembrane helix^15^, respectively. This stepwise process is consistent with our previous time-lapse video microscopic study^10^,^12^. A bright spot of GFP-M-Sec signal first appears on the plasma membrane and, subsequently, a short membrane nanotube gradually extends outwards from this spot. This short nanotube eventually contacts the plasma membrane of a neighboring cell. GFP-M-Sec could also be detected in the nucleus, although we do not know the physiological significance of these nuclear M-Sec; importance of the nuclear M-Sec should be carefully interpreted, since exogenously introduced and overly expressed molecules often mislocalized to the nucleus, especially in the case of GFP-fusion proteins.

We showed that the M-Sec N-terminal polybasic region is responsible for the interaction with phosphoinositides, indicating that the recruitment of M-Sec to the plasma membrane is mediated by electrostatic interactions between anionic lipids and cationic amino acids. Such electrostatic interactions mediate reversible protein-membrane interactions, and can be modulated by addition or removal of phosphates^37^,^38^. A recent phosphoproteomics study has revealed that, following LPS stimulation, the vicinity of the N-terminal polybasic region of M-Sec is phosphorylated in the polo-like serine/threonine kinase-dependent manner^39^. The N-terminal phosphorylation may retain M-sec in the cytoplasm, and dephosphorylation may enhance its recruitment to the plasma membrane. TNT formation is rapidly induced by apoptotic signaling^40^ and oxidative stress^41^ (within 30 min and 2 hours, respectively). The N-terminus of M-Sec might function as a switch for initiating membrane deformation. This region is predicted to be α-helical but disordered (or highly mobile). Accordingly, the corresponding electron density was invisible in the present crystallographic analysis. Alternatively, specific recognition of PI(4,5)P_2_ and/or PI(3,4,5)P_3_ would require the pre-existing binding pocket which likely involves the N-terminal α-helix region. We favor the idea that the N-terminal region of M-Sec can be helically folded upon binding to the head group of PI(4,5)P_2_ to form the binding pocket, such as occurs with the Epsin1 ENTH domain^42^.

The crystal structure of M-Sec consists of five helix-bundle domains, four of which are arranged in a manner similar to those of mouse and yeast Exo70 subunits (PDB IDs: 2PFV and 2BLE, respectively). Both M-Sec and Exo70 have a conserved, positively charged surface and can induce long membrane protrusions^43^,^44^ when overexpressed in mammalian cells. However, the underlying mechanisms seem different between M-Sec and Exo70. Exo70 can induce long membrane protrusions independently of the exocyst complex^43^. Moreover, Exo70 directly binds to the Arp2/3 complex, which then stimulates actin polymerization and pushes the plasma membrane to generate surface protrusions^43^,^44^. The positively charged surface of Exo70 is responsible for binding to PI(4,5)P_2_^35^ and the Arp2/3 complex^44^. On the other hand, M-Sec by itself can induce short membrane deformation but requires the Ral/exocyst complex to generate long protrusions. The positively charged surface of M-Sec is responsible for the interaction with RalA but not with PI(4,5)P_2_. RalA is a multifunctional small GTPase involved in cell proliferation^45^, membrane trafficking^46^, transcriptional regulation^47^, cell migration^48^, and actin cytoskeletal remodeling^49^. Because TNT formation is accompanied by actin cytoskeletal remodeling, M-Sec likely activates signaling pathways of RalA-mediated actin cytoskeletal remodeling on the plasma membrane to elongate membrane protrusion via its downstream effectors such as filamin, Cdc42 and the exocyst complex.

Although we confirmed that the interaction between active RalA and the positively charged surface on domains D and E of M-Sec is critical for membrane extension during TNT formation, we have not succeeded in detecting the direct interaction between M-Sec and RalA by *in vitro* binding assays with purified M-Sec and RalA that were produced in *E. coli*. This leaves open the possibility of indirect binding through other molecule(s), or involvement of posttranslational modification and/or co-factors. Clarification of this mechanism thus remains an important goal for future efforts to further understand how M-Sec regulates TNT formation in cooperation with RalA and the exocyst complex.

## Methods

### Plasmids and site-directed mutagenesis

M-Sec cDNA (GenBank accession No. NM_009396.1) was generated from mouse splenic macrophage mRNA by reverse transcriptase polymerase chain reaction and inserted into pBluescript II KS (+) (Agilent Technologies) as described previously^10^. The *Bam*HI*-Sal*I fragment was subcloned into the mammalian expression vector pEGFP-C1 (Clontech Laboratories, Inc.), pStrawberry-C1^10^ or a bacterial expression vector pGEX4T-2 or pGEX6P-1 (GE Healthcare) to produce recombinant glutathione S-transferase (GST)-fusion proteins. Deletion and point mutants of M-Sec were generated using KOD-Plus-Mutagenesis kit (Toyobo Life Science) following the manufacturer’s instruction. The primers used for the mutagenesis are listed in Supplementary Table S1. Products were sequenced using an ABI Prism 3100-Avant genetic analyzer (Applied Biosystems). We have optimized transfection conditions for the individual plasmid constructs by checking protein expression levels with Western blotting analysis (Supplemental Fig. S6). The plasmids encoding PLCδ-PH-GFP and Akt-PH-GFP were obtained from Addgene (plasmid No. 21179 and 18836, respectively).

### Cell culture, transfection, immunocytochemistry and fluorescence microscopy

HeLa cells were obtained from the American Type Culture Collection. HEK293A cells were purchased from Life Technologies. Cells were cultured at 37 °C with 5% CO_2_ in Dulbecco’s modified Eagle Medium supplemented with 10% fetal bovine serum.

HeLa cells were cultured on coverslip-bottom 24-well plates pre-coated with 1% gelatin (Wako Biochemicals), and were transfected with the plasmid coding GFP-M-Sec or GFP-M-Sec mutants using the Lipofectamine LTX (Life Technologies). The transfected cells were further cultured overnight, and then washed twice with ice-cold phosphate buffered saline (PBS). The washed cells were treated with biotinylated wheat germ agglutinin (WGA) (Vector Labs) on ice for 10 min for staining the plasma membrane. The cells were fixed with 4% paraformaldehyde for 15 min at room temperature. To visualize the biotinylated WGA, the fixed cells were washed with PBS and then treated with streptavidin-Alexa647 (Life Technologies) for 30 min.

Image stacks covering the whole cellular volume were acquired using a laser scanning confocal microscope, FV300 (Olympus). The “TNT-forming cells” in Figs 1d and 2b are the cells connected with two or more cells by WGA-positive thin membrane structures that do not touch the substratum. The TNT structures were detected from sliced images reconstructed from consecutive confocal images by using ImageJ software. The “GFP-positive membrane protrusion” is a GFP-positive structure that includes the TNT structure as well as a membrane protrusion whose leading edge is free and does not connect with another cell. The length of the GFP-positive membrane protrusion was measured by using ImageJ software (http://rsb.info.nih.gov/ij/) in Fig. 6.

For immunocytochemistry analysis of PI(4,5)P_2_ or PI(3,4,5)P_3_, cells were fixed with 4% paraformaldehyde plus 0.1% glutaraldehyde in PBS for 15 min at room temperature. After fixation, all procedures were performed at 4 °C. For blocking unreacted aldehyde groups, cells were washed with PBS containing 50 mM NH_4_Cl and 20 mM glycine for five times, and then the cells were incubated with anti-PI(4,5)P_2_ (Life Technologies) or anti-PI(3,4,5)P_3_ monoclonal antibodies (Life Technologies) diluted in PBS containing 0.2% saponin and 0.2% BSA, followed by Cy3-conjugated anti-mouse IgM antibodies (Jackson Immunoresearch). The cells were observed with confocal microscopy. Colocalization efficiencies of M-Sec with PI(4,5)P_2_ or PI(3,4,5)P_3_ were measured using ImageJ software.

### Cell fractionation

HeLa cells transfected with expression vectors encoding GFP-M-Sec or the GFP-M-Sec mutants were washed with PBS, harvested in chilled 50 mM Tris-HCl (pH 8.0) containing 1 mM dithiothreitol (DTT), 150 mM NaCl, 2.5% sucrose and protease inhibitor cocktail (Roche diagnostics), and homogenized with a pestle for 10 times in a microcentrifuge tube. After 1,000 × g centrifugation of the homogenate, the supernatant (postnuclear supernatant: PNS) was ultracentrifuged for 60 min at 100,000 × g (TLA100 rotor; Beckman) at 4 °C. After taking the supernatant as the soluble cytosol fraction, the pellet was resuspended in the same homogenization buffer and ultracentrifuged once more with using the same conditions. The second supernatant was discarded and then the resulting pellet was suspended in the homogenization buffer with 1% Triton X-100, and then ultracentrifuged with using the same conditions. This supernatant was used as the membrane fraction. Each fraction was diluted to achieve equivalent volumes and subjected to Western blotting analysis. Each antibody was diluted as below for immunoblotting: anti-MCT1 antibody (Biogenesis; 100-fold dilution), anti-Rpt4 antibody (Enzo Life science; 1,000-fold dilution) and anti-GFP antibody (Frontier Science; 5,000-fold dilution).

### Liposome co-sedimentation assay

We induced the overexpression of the various M-Sec proteins in the *Escherichia coli* strain BL21 (DE3) (Life Technologies) by addition of 0.1 mM isopropyl-β-D-thiogalactopyranoside (IPTG) at 25 °C. For the purification of M-Sec proteins for the liposome cosedimentation assay, the harvested bacterial cells were resuspended in 50 mM Tris-HCl (pH 8.0) containing 5 mM DTT, 150 mM NaCl and 2.5% sucrose. We lysed the suspension with 0.1 g/L lysozyme (Wako Biochemicals) and 0.25 unit/μL turbonuclease (Accelagen) at room temperature for 30 min and sonication. The clarified lysates were then loaded onto a Glutathione Sepharose FF column (GE Healthcare), and the GST fusion proteins were eluted with 50 mM Tris-HCl buffer (pH 8.0) containing 150 mM NaCl, 1 mM DTT, 2.5% sucrose and 15 mM reduced glutathione.

Synthetic liposomes contained 20% phosphatidylethanolamine, 45% phosphatidylcholine and 30% phosphatidylserine supplemented with 5% respective phophatidylinositols (molecular weight ratio), or total liver lipids were prepared as described previously^36^. All lipids were purchased from Avanti Polar lipids. Liposomes were resuspended at concentration of 0.6 g/L in 50 mM Tris-HCl (pH 8.0), 1 mM DTT, 150 mM NaCl and 2.5% sucrose and were sized by extrusion using an Avanti Mini-extruder (Avanti Polar Lipids) with a 0.1-μm-pore size filter. For sedimentation assays, each protein was incubated with the liposomes for several minutes before sedimentation at 30,000 rpm for 15 min in a Beckman TLA100.2 rotor. Vesicle pellets were suspended in SDS sample buffer and fractionated by SDS-PAGE. Co-precipitated proteins were identified by Coomassie Brilliant Blue staining or Western blotting and quantified by densitometry with ImageJ software^50^.

### Crystallography

The full-length or N-terminally truncated M-Sec were produced as the N-terminal GST fusion protein in *E. coli* strain Rosseta^TM^ (DE3) (Invitrogen). Overexpression was induced by 0.1 mM IPTG. The cells were suspended in phosphate buffer (pH 7.4) containing 1 mM DTT, 300 mM NaCl and 1 mM phenylmethylsulfonyl fluoride (PMSF), and then disrupted by sonication. After centrifugation, the supernatant was loaded onto a Glutathione Sepharose FF column (GE Healthcare), and the GST fusion protein was eluted with 50 mM Tris-HCl buffer (pH 8.0) containing 150 mM NaCl, 1 mM DTT and 15 mM reduced glutathione. The GST tag was cleaved by HRV3C protease (GE Healthcare). After purification with a ResourceQ anion exchange column (GE Healthcare), the fractions containing the eluted protein was loaded onto a Glutathione Sepharose FF column to completely remove the cleaved GST tag. The flow through fraction was loaded onto Superdex200 16/60 (prep grade) column (GE Healthcare) with 10 mM Tris-HCl buffer (pH 8.0) containing 150 mM NaCl and 5 mM β-mercaptoethanol. The fractions containing the protein were used for crystallization. Selenomethionine-labeled protein was prepared in the same way as the native protein, except that *E. coli* strain B834 (DE3) and Core^TM^ medium (Wako Biochemicals) supplemented with 30 mg/L of L-selenomethionine (Nacalai Tesque) were used for the culture.

The purified M-Sec samples were concentrated to 5–10 g/L by using Amicon Ultra 15 (Millipore). More than 500 crystallization conditions (Hampton Research) were screened by the sitting drop vapor diffusion method at 20 °C with a Mosquito liquid-handling robot (TTP Lab Tech). The initial hits were further optimized. The best crystals of the full-length protein were obtained by mixing 1 μL of the protein solution with an equal volume of reservoir solution containing 0.1 M HEPES-Na (pH 7.0), 1–4% (w/v) polyethylene glycol (PEG) 8000, 8% (v/v) ethylene glycol, which was equilibrated against 500 μL of the reservoir solution. The best crystals of the N-terminally truncated protein were obtained in the same way, except that the reservoir solution containing 5–7% (w/v) PEG 8000 was used.

Diffraction data sets were collected at beamline BL41XU in SPring-8 (Hyogo, Japan), and processed with HKL2000^51^ and CCP4 program suite^52^. Heavy-atom site search, phase calculation and density improvement of the SAD data set were conducted by SHELX97^53^, SHARP^54^ and SOLOMON/DM under the control of autoSHARP^55^. The atomic model was built with the program COOT^56^ and refined to 3.0 Å resolution by using the program Phenix^57^. Data collection, phasing and refinement statistics are shown in Supplementary Table S2. All molecular graphics were prepared with the program PyMOL (DeLano Scientific; www.pymol.org).

### GST pull-down experiments with recombinant RalA protein

HEK293A cells transfected with expression vectors encoding GFP-M-Sec or GFP-M-Sec mutants were washed with PBS and lysed in lysis buffer containing 25 mM Tris-HCl (pH 7.4), 1 mM EGTA, 150 mM NaCl, 2.5 mM MgCl_2_, 1% Triton X-100, 2.5% sucrose and protease inhibitor cocktail (Roche diagnostics). The cell lysates were centrifuged at 1,000 × g for 10 min at 4 °C. The supernatants were precleared by the addition of GSH-Sepharose for 30 min, pelleted at 100 × g for 5 min at 4 °C. GST-RalA beads were loaded with guanine nucleotides using established methods^58^,^59^. A volume of GSH beads containing 10 μg of GST-RalA was incubated with an equal volume of 20 mM Tris-HCl (pH 7.4), 10 mM EDTA, 25 mM NaCl, and 1 mM GDP or 200 μM GTPγS at 37 °C for 30 min. The buffer was adjusted to 10 mM MgCl_2_, then the tissue lysates were added and incubated at 4 °C for 2 hours. The beads were isolated by centrifugation at 100 × g for 5 min, and washed three times with ice-cold lysis buffer followed by three washes with 20 mM Tris-HCl (pH 7.4) containing 2.5 mM MgCl_2_. Samples were run on SDS-PAGE and subjected to Western blotting analysis. Each antibody was diluted as follows for immunoblotting: anti-Sec8 antibody (Stresgen; 1,000-fold dilution) and anti-GFP antibody (Frontier Science; 5,000-fold dilution).

### Statistics

Differences between the two groups were analyzed by Student’s *t*-test. Differences for multiple group comparisons were analyzed by a one-way analysis of variance and Tukey-Kramer multiple comparison tests. All statistical analyses were conducted using R software (http://www.r-proj- ect.org/).

## Additional Information

**Accession codes**: Coordinates and structure factors of M-Sec have been deposited in the Protein Data Bank under accession code 5B86.

**How to cite this article**: Kimura, S. *et al.* Distinct Roles for the N- and C-terminal Regions of M-Sec in Plasma Membrane Deformation during Tunneling Nanotube Formation. *Sci. Rep.* **6**, 33548; doi: 10.1038/srep33548 (2016).

## Supplementary Material

Supplementary Information
