# Supplementary material for: Distinct Roles for the N- and C-terminal Regions of M-Sec in Plasma Membrane Deformation during Tunneling Nanotube Formation
Source: Sci Rep. 2016 Sep 15;6:33548. doi: 10.1038/srep33548 (PMC5024327; doi:10.1038/srep33548)

Distinct Roles for the N- and C-terminal Regions of M-Sec in Plasma Membrane Deformation during Tunneling Nanotube Formation

Shunsuke Kimura, Masami Yamashita, Megumi Yamakami-Kimura, Yusuke Sato, Atsushi Yamagata, Yoshihiro Kobashigawa, Fuyuhiko Inagaki, Takako Amada, Koji Hase, Toshihiko Iwanaga, Hiroshi Ohno, and Shuya Fukai

Supplementary Information

Supplementary Figure Legends

Supplementary Figure S1

Lipid co-sedimentation assays of M-Sec mutants with $PI(4,5)P_2$. GST-M-Sec mutant proteins were incubated with liposomes containing $PI(4,5)P_2$. The proteins co-precipitated with liposomes were visualized by Coomassie Brilliant Blue staining. The bound proteins were quantified by densitometry. The data is a representative of three independent experiments.

Supplementary Figure S2

Fluorescent microscopic analysis using $PI(4,5)P_2$ and $PI(3,4,5)P_3$ probes with Strawberry-M-Sec.

HeLa cells were transfected with expression vectors encoding PLCδ-PH-GFP (a) or Akt-PH-GFP (b) together with a vector encoding Strawberry-M-Sec. PLCδ-PH-GFP was used as $PI(4,5)P_2$ probe, and Akt-PH-GFP was used as $PI(3,4,5)P_3$ probe..

PLCδ-PH-GFP was localized in M-Sec-positive membrane protrusions, whereas the Akt-PH-GFP fluorescent signal was very weak in these membrane structures. Each lower panel is an enlarged image from the boxed area in its corresponding upper panel. Scale bars represent 10 μm.

Supplementary Figure S3

$PI(4,5)P_2$ is localized in TNT structure in Raw264.7 cells.

Raw264.7 cells were fixed with 4% formaldehyde plus 0.1% glutaraldehyde, and then subjected to immunocytochemistry by using anti-$PI(4,5)P_2$. Bar represents 20 μm.

Supplementary Figure S4

Structural comparison of mouse M-Sec with *S. cerevisiae* Sec6 (PDB 2FJI residues 411–805 out of 805), *Homo sapiens* COG4 (PDB 3HR0, residues 525-785 out of 785), *S. cerevisiae* Dsl1 (PDB 3K8P, residues 333–684 out of 754), *S. cerevisiae* Vps53 (PDB 3NS4, residue 554-822), *S. cerevisiae* Myo4 (PDB 3MMI, residues 1091-1471 out of 1472), *S. cerevisiae* Tip20 (PDB 3FHN, residues 1-701 out of 701) and *S. cerevisiae* Exo70 (PDB 2B7M, residues 67–623 out of 623). All structures were aligned based on the superposition of domains C–E. Pairwise alignment was performed with the program DaliLite to match each of the structures to M-Sec with Z scores of 25.1 (M-Sec-Sec6p), 12.1 (M-Sec-Cog4), 14.0 (M-Sec-Dsl1), 7.6 (M-Sec-Vps53), 14.5 (M-Sec-Myo4), 12.8 (M-Sec-Tip20) and 11.4 (M-Sec–Exo70).

Supplementary Figure S5

(a) Multiple amino acid sequence alignment of the C-terminal region of M-Sec. Amino-acid sequences from six mammalian species were aligned using ClustalX software (version 2.0.10; www.clustal.org/clustal2). Lysine and arginine residues are highlighted by a gray background. Asterisks indicate the residues mutated in this study. (b) Cell fractionation assays of M-Sec mutants. HeLa cells were transfected with the expression vectors encoding the indicated proteins. Cell fractionations and western blotting analyses were performed as described in the online *Methods*. The double and quadruple M-Sec mutants were detected in the membrane fraction.

Supplementary Figure S6

Estimation of protein expression level by each plasmid construct with Western blotting. HeLa cells were transfected with the indicated constructs and analyzed for the expression of each protein by Western blotting using the anti-GFP-antibody. Rpt4 was used as a loading control.

Supplementary Table S1 The primer pairs used for the mutagenesis

Mutant name	Forward Primer	Reverse Primer
107-650	5'-atgagcaacgaggagctggtgtgg-3'	5'-ggatctgagtccggacttgta-3'
483-650	5'-aatccagttgagaccctggagg-3'	5'-ggatctgagtccggacttgta-3'
1-482	5'-tagataactgatcataatcagcc-3'	5'-ggcctccagtgccgccgcc-3'
1-173	5'-tagataactgatcataatcagcc-3'	5'-tgcccaacgggtctgtgtg-3'
ΔK1	5'-ggactggccaacatgttcagtg-3'	5'-cgcagactcctcctccccatc-3'
ΔK2	5'-gaccagcccagattatcagatctgg-3'	5'-ggtgaagacactgaacatgttggcc-3'
ΔK1&K2	5'-ggactggccaacatgttcagtg-3'	5'-ggtgaagacactgaacatgttggcc-3'
Quadruple	5'-GCCacggccgagcagcagcagcag-3'	5'-gaggaccagGGCGGCGGCgcagagcc-3'
Double	5'-gtttGCCaagttcacacagacccgttgg-3'	5'-agcggGGCcaggtccaaaaataggctctgg-3'

Substituted nucleotides in the primers are shown in upper case.

Supplementary Table S2 Data collection, phasing and refinement statistics

	M-Sec (high resolution)	M-Sec (SeMet)
**Data Collection**		
Space group	$P2_12_12_1$	
Unit cell parameter	a = 91.4 Å, b = 107.8 Å, c = 229.6 Å	a = 91.1 Å, b = 108.0 Å, c = 230.3 Å
Wavelength (Å)	0.97904	
Resolution (Å)	50.0-3.0 (3.05-3.0)	50.0-3.37 (3.43-3.37)
Unique reflections	43,284	32,800
Total reflections	131,314	472,356
Completeness (%)	95.9 (90.1)	99.9 (99.7)
I / $\sigma(I)$	6.0 (1.4)	14.3 (4.3)
R_{sym}	0.159 (0.520)	0.195 (0.548)
**Phasing**		
Number of Se sites		18
Phasing power		0.374
R_{cullis}		0.967
FOM (acentric/centric)		0.195/0.095
**Refinement**		
Number of atoms: protein / water	9,266 / 49	
Rmsd bond length (Å)	0.010	
Rmsd bond angle (°)	1.481	
Average B factors (Å^2)	79.69	
Residues in core region (%)	88.3	
Residues in additionally allowed region (%)	10.7	
Residues in generously allowed region (%)	1.0	
Residues in disallowed region (%)	0.0	
R_{work}, R_{free}	0.229, 0.292	

The numbers in parentheses are for the highest resolution shell.

$R_{sym} = \Sigma|I_{avg} - I_i|/\Sigma Ii$.

$R_{cullis} = \Sigma||\mathbf{F}_{PH} - \mathbf{F}_P| - |\mathbf{F}_{H(calc)}|| / \Sigma|\mathbf{F}_{PH}|$.

$R_{work} = \Sigma|F_o - F_c|/\Sigma F_o$ for reflections of the working set.

$R_{free} = \Sigma|F_o - F_c|/\Sigma F_o$ for reflections of the test set (5% of total unique reflections).

Supplementary Figure S1

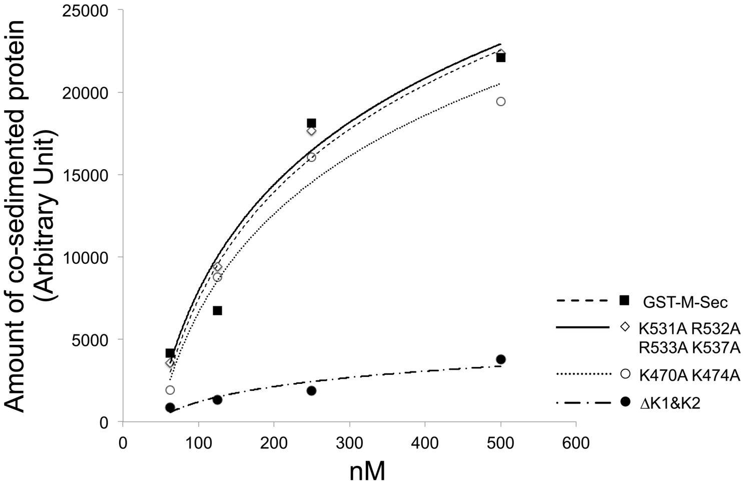

Supplementary Figure S2

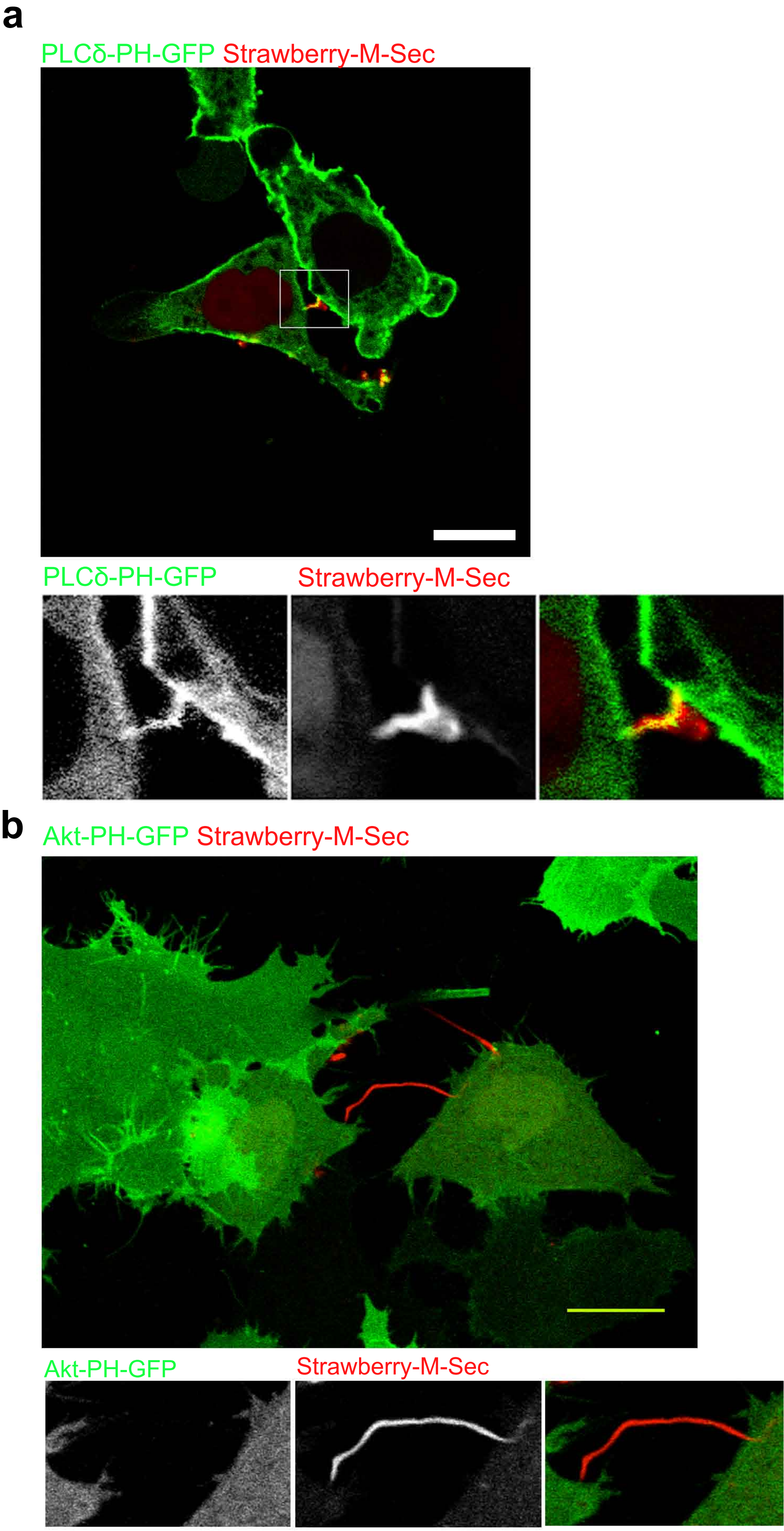

Supplementary Figure S3

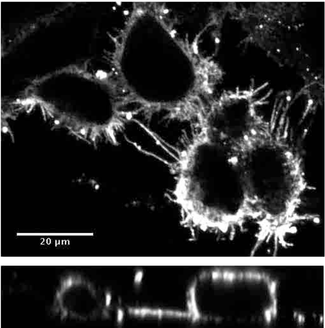

Supplementary Figure S4

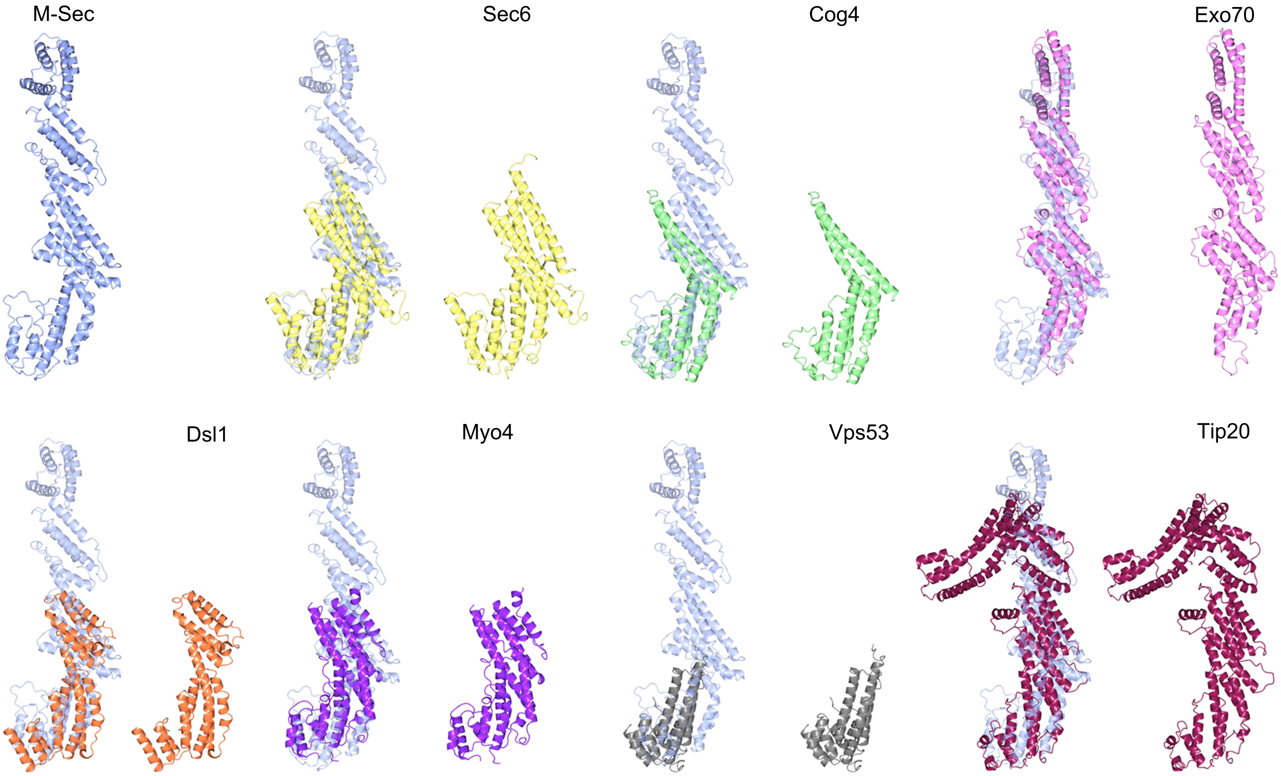

Supplementary Figure S5

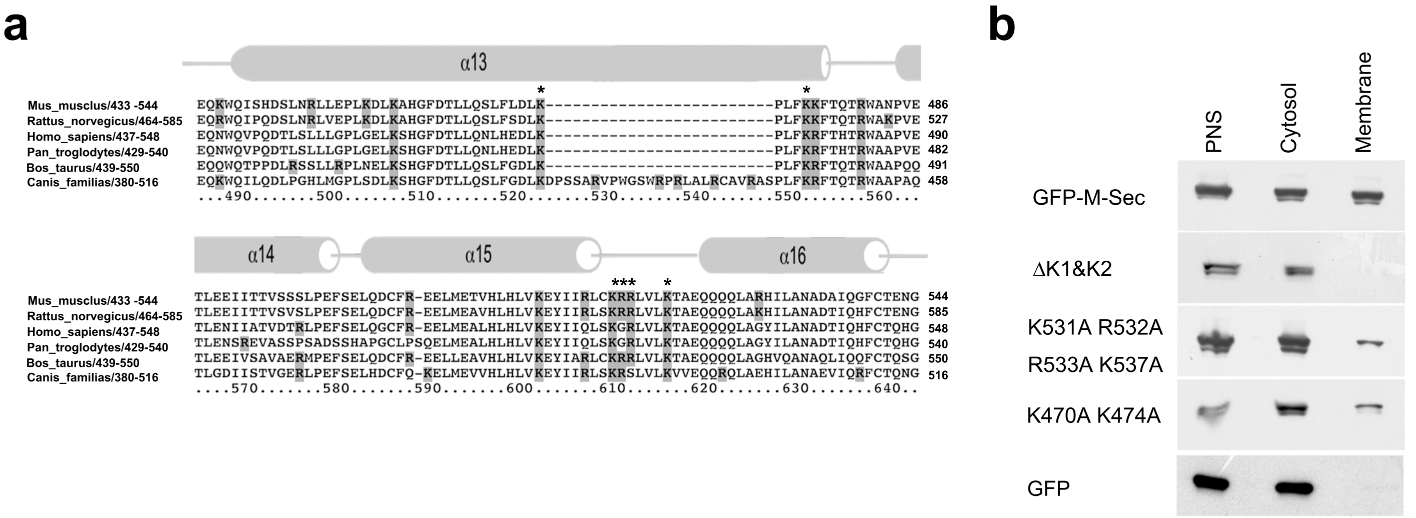

Supplementary Figure S6

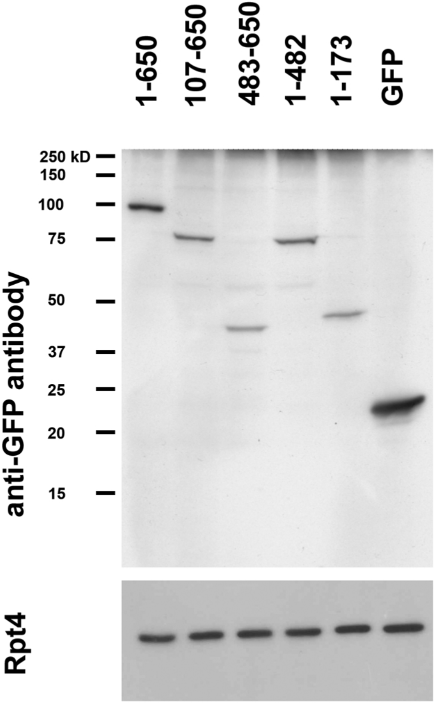

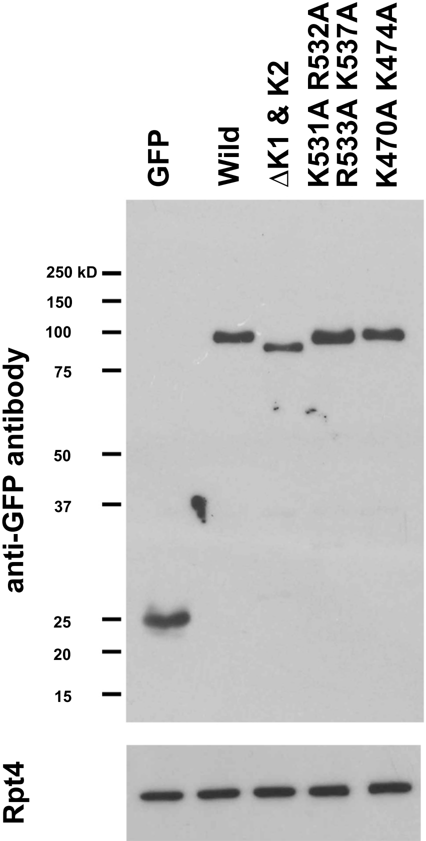

Supplement: Supplementary Information [file srep33548-s1.pdf]
